# Hematological profiles and preventive-practice characteristics among patients with podoconiosis: A case-control study in Musanze District, Rwanda

**DOI:** 10.1371/journal.pntd.0014652

**Published:** 2026-08-17

**Authors:** Hiberte Migabo, Diogene Rwayitare, Jean Paul Bikorimana, Alexis Nshimiyimana, Consolee Uwamahoro, Kazi Tani Sarra Latifa, Laurent Charlet, Thierry Habyarimana

**Affiliations:** 1 Department of Biomedical Laboratory Sciences, INES-Ruhengeri, Musanze, Rwanda; 2 Centre for Gender Studies, College of Art and Social Sciences, University of Rwanda, Kigali, Rwanda; 3 Heart and Sole Action, Musanze, Rwanda; 4 Institute of Earth Science (ISTerre), Université Grenoble Alpes, Université Savoie Mont Blanc, CNRS, IRD, Université Gustave Eiffel, Grenoble, France; Huazhong University of Science and Technology Tongji Medical College, CHINA

## Abstract

**Background:**

Podoconiosis is a chronic, disfiguring, non-filarial form of lymphedema caused by prolonged barefoot exposure to irritant soil. An estimated four million people are affected globally, with cases documented in more than 32 countries, 18 of which are in Africa. In Rwanda, the disease affects an estimated 68.5 per 100,000 people across all districts. While literature on podoconiosis continues to grow, its hematological abnormalities remain relatively unexplored.

**Methodology/ Principal findings:**

This case-control study aimed to investigate hematological profile in podoconiosis patients and associated factors. A total of 119 participants (50 cases and 69 controls), above 18 years old were enrolled. Data were collected at Heart and Sole Action clinic in Musanze, and laboratory analyses were conducted at INES-Ruhengeri Hematology Laboratory. Most participants were female, engaged in farming, and had no formal education. Podoconiosis patients were older than controls and demonstrated poor foot hygiene, inconsistent shoe use, and limited disease knowledge, with 40% reporting a family history and an average disease onset at 21.06 ± 13.15 years. Although the values for each patient fell within the normal reference range and no morphological abnormalities were observed on peripheral blood smears, quantitative differences were observed between cases and controls and revealed significantly reduced lymphocyte percentages, RBC indices (RBC, hemoglobin, hematocrit, MCHC).

**Conclusion/Significance:**

These findings indicate systemic hematological difference likely linked to chronic immune activation, highlighting the importance of integrating hematological monitoring and targeted health education into podoconiosis prevention and management strategies. Future studies should also explore the link between blood alterations and disease severity.

## Author summary

Podoconiosis is a long-term disease that leads to leg swelling and disability, mainly affecting individuals who walk barefoot on irritant soil. It carries significant social and economic burdens and is considered a neglected disease. While research has explored its causes and clinical features, gaps remain regarding its impact on blood. In this study, we assessed risks and analysed blood samples from individuals with and without podoconiosis. Patients with the disease showed changes in various blood components, such as reduced levels of red blood cells and certain white blood cells, possibly indicating immune system activation due to the disease. Although the blood results remained within normal ranges, the observed differences point to possible underlying systemic effects. Notably, more than 94% of podoconiosis patients developed the condition at an early age, underscoring the need for preventive measures in childhood and adolescence. Further research is warranted to explore additional hematological factors and to investigate biological mechanisms behind these blood changes.

## Introduction

Podoconiosis is a chronic, disfiguring, non-filarial form of lymphedema caused by prolonged barefoot exposure to irritant mineral particles found in volcanic soil [1,2]. These particles penetrate the skin of the feet and trigger an inflammatory immune response [3]. However, research suggests that exposure to soils alone does not guarantee disease development, indicating that genetic factors influence individual susceptibility. This supports the understanding that the condition results from an interaction between genetic predisposition and environmental exposure, with no specific biological agent identified as the etiological factor [4]. In the third decade of life, the condition commonly appears in subsistence farmers and is characterised by bilateral, asymmetric enlargement of the lower limbs below the knees [5].

Podoconiosis represents significant public health concern, particularly in tropical highland regions. Globally, at least four million people are believed to be affected by podoconiosis, with cases documented in more than 32 countries:18 in Africa, 3 in Asia, and 11 in Latin America [6]. Africa bears the greatest burden of disease, with more than 114 million people living in environmentally suitable areas and at risk of developing it [8]. However, its prevalence varies widely between countries and within regions, often correlating with factors such as poverty levels, soil composition, altitude, and access to protective measures such as footwear [7]. Podoconiosis shows the highest environmental susceptibility in East Africa, as it affects around 80% of more than 100 million people with a risk of developing the condition [8]. Ethiopia carries the largest documented burden, with prevalence estimates of up to 4%, affecting approximately 0.5 to 1 million people and placing an additional 19.2 million individuals (22–24% of the population) at risk of developing the disease [11]. In Cameroon, approximately 22.3% of the population lives in environmentally suitable areas, with an estimated rural prevalence of 4.9%, while prevalence rates of 3.87% in Kenya, 4.52% in Uganda, and 0.99% in Burundi further demonstrate its widespread occurrence.

In Rwanda, podoconiosis is also widespread, with a high prevalence in the Northern and Western provinces. It is estimated that more than 6,000 people are living with podoconiosis across Rwanda, with an overall prevalence of 68.5 cases per 100,000 population, varying across districts [9].

Podoconiosis patients often face physical pain, psychological distress, and economic hardship, including job loss and reduced productivity [10]. They are frequently excluded from social institutions and community life, and many experience stigma and family rejection. Additionally, depression is significantly more common among affected individuals [11,12].

Podoconiosis involves continuous immune activation, inflammatory processes [1], and excessive or chronic inflammation is a contributing factor to numerous diseases of hematopoietic and lymphatic systems [13]. Normally, inflammation plays a crucial role in regulating hematopoietic stem cell (HSC) function, influencing their fate, proliferation, and differentiation. Still, prolonged inflammation alters the bone marrow niche, disrupting the supportive environment required for balanced hematopoiesis [14]. Chronic inflammatory diseases significantly impact hematopoiesis, leading to conditions such as anemia of chronic disease (ACD), thrombocytosis, and leukocytosis. Additionally, prolonged inflammatory states can predispose individuals to myelodysplastic syndromes and certain leukemia due to persistent immune activation and oxidative stress [15].

Research suggests that podoconiosis, traditionally associated with localised lymphatic inflammation in the lower limbs, may also affect systemic hematological parameters, and variations in the hematological parameters could, therefore, have significant clinical implications [16]. Studies have found significant alterations in blood parameters among affected individuals, such as changes in red cell and platelet indices (e.g., MPV, PCT, PDW, MCV, RDW-SD), suggesting underlying subclinical disruptions [10]. Similarly, another study observed significant changes with reduced levels of white blood cells (especially neutrophils and eosinophils), hemoglobin, and red blood cell indices (MCH, MCHC). Conversely, elevated lymphocyte and monocyte counts indicate possible immune dysregulation and chronic inflammation in podoconiosis patients [17].

The burden of these hematological abnormalities may contribute to increased susceptibility to infections and other risks. Despite these contributions, data on the hematological profiles of podoconiosis patients in Rwanda are lacking and understanding these alterations is essential not only for improving clinical management but also for informing policy decisions regarding podoconiosis surveillance and prevention strategies. Therefore, this study aimed to assess and compare hematological parameters and peripheral blood smear morphology between podoconiosis patients and healthy controls in Musanze District, Rwanda. The study hypothesized that podoconiosis patients would exhibit significant alterations in hematological parameters compared with healthy controls.

## Methods

### Ethics statement

Prior to the study, official approval was obtained from the Institut d’Enseignement Supérieur de Ruhengeri (INES-Ruhengeri) [INES-IRB/2025/01/29], Musanze District [1499/07.04.03], and Heart and Sole Action (HASA), ensuring adherence to research ethics. The study was conducted in accordance with the principles of the declaration of Helsinki. Participants provided written informed consent before enrollment. All information was handled confidentially to protect participants’ privacy. Furthermore, to ensure anonymity and confidentiality of results, the samples were labelled using coded identifiers.

### Study area

The study was conducted in Musanze District, located in the Northern Province of Rwanda. This district was selected due to its documented prevalence of podoconiosis. Data collection took place specifically at Heart and Sole Action (HASA) Musanze clinic, a non-governmental organisation dedicated to supporting individuals affected by podoconiosis across Rwanda. Following data and sample collection, laboratory analyses were subsequently performed at the INES-Ruhengeri Hematology laboratory.

### Study design and study population

This study adopted a unmatched case-control laboratory-based approach. The study population included adults above 18 years old. The study was conducted from July 2024 to August 2025. Cases were individuals with clinically confirmed podoconiosis, while controls were healthy participants from the same geographic area with no history or clinical signs of podoconiosis. The control group included neighbors and unaffected family members of podoconiosis patients to ensure comparability in environmental exposures, which can influence hematological parameters. Potential controls were additionally screened for underlying hematological or systemic conditions that could affect hematological parameters, including liver disease, kidney disease, anemia, and malaria; individuals with such abnormalities were excluded to ensure comparability with podoconiosis cases.

In addition, all participants should have lived in Musanze District for a minimum of two years, be willing to participate and provide written informed consent. Individuals with lymphedema caused by conditions other than podoconiosis, pregnant or lactating women due to physiological hematological changes, participants with other chronic disorders and anyone who declined participation were excluded.

### Sample size

Participants were selected using convenience sampling based on their availability and willingness to participate during the study period. A total of 119 participants attending HASA Musanze Clinic were enrolled in the study, including 50 podoconiosis patients (cases) and 69 healthy individuals (controls). Participants were recruited from 29 April to 11 June 2025. All eligible patients attending the clinic during this period were invited to participate. Non-participation was mainly due to refusal to consent, limited time, poor health, or failure to meet the eligibility criteria. Because no complete patient register was available following service decentralisation, no formal random-sampling procedure was used.

### Data collection

A structured and validated questionnaire was administered to gather relevant information. The questionnaire consisted of three sections: sociodemographic characteristics, environmental exposure and disease history. Only participants in the case group responded to the section on disease history. To ensure accuracy, participants were guided through the questionnaire, which was administered through face-to-face interviews conducted in Kinyarwanda (mother tongue).

### Blood sample collection

Blood samples were collected aseptically from each participant by trained laboratory personnel following standard venipuncture procedures. Collections were performed in the morning (8:00–11:00 AM) to reduce diurnal variation. Approximately 3 ml of venous blood was collected from each participant into a properly labelled anticoagulated (EDTA) tubes for hematological analysis. Proper labelling with a unique participant code was used.

### Hematological analysis

Hematological parameters were measured using an automated hematology analyser, Humacount 60^TS^ (Germany). The hematological parameters assessed included hemoglobin (Hb), red blood cell count (RBC), white blood cell count (WBC), platelet count and platelet indices, red blood cell indices (mean corpuscular volume [MCV], mean corpuscular hemoglobin [MCH], mean corpuscular hemoglobin concentration [MCHC], and red cell distribution width [RDW]), and differential leukocyte counts. Peripheral blood smears were prepared immediately after collection**,** stained using Romanowsky stain (May-Grünwald Giemsa), and examined microscopically to assess morphological changes and differential leucocytes count. All analyses were conducted in accordance with standard operating procedures, and quality control materials were run to ensure accuracy and precision.

### Statistical analysis

Data were analysed using SPSS version 26. Descriptive statistics were computed to summarise the data. The primary variables were a range of hematological parameters measured as continuous variables. Assumptions of normality and equal variance were tested using the Shapiro-Wilk test and Levene’s test, respectively. Depending on the distribution of the data, either an independent t-test or the Mann-Whitney U test was used to compare hematological parameters between podoconiosis patients and controls. To account for multiple comparisons across hematological parameters, p-values were adjusted using the Benjamini–Hochberg false discovery rate (FDR) method, A significance threshold was set at *p* < 0.05. Effect sizes for non-parametric comparisons were calculated using the rank-biserial correlation coefficient (r = Z/√N) and interpreted as small (0.1), moderate (0.3), or large (0.5). Factors related to podoconiosis knowledge and preventive practices were summarised descriptively and compared between cases and controls.

## Results

### Sociodemographic characteristics

A total of 119 individuals, including 50 podoconiosis cases and 69 healthy controls from the same geographic area, were included in the study. Among the control group, 35 participants were family members of podoconiosis patients, while 34 were neighbors from the same communities. The majority of participants were female (84%), with a similar gender distribution observed in both groups. The mean age of podoconiosis cases was 52.9 ± 15.21 years, which was notably higher than control group, whose mean age was 42.35 ± 13.73 years. Additionally, most participants in both groups were married, engaged in farming, and had no formal education (Table 1).

**Table 1:** Sociodemographic characteristics of the study population.

Variable	Category	Cases (%)	Control (%)
Gender	Female	42 (84)	58 (84)
	Male	8 (16)	11 (16)
Age group (years)	18–30	5 (10)	17 (25)
	31–40	7 (14)	15 (22)
	41–50	7 (14)	16 (23)
	51–60	13 (26)	14 (20)
	61 and above	18 (38)	7 (10)
Marital status	Single	9 (18)	10 (14)
	Married	23 (46)	42 (61)
	Divorced	8 (16)	4 (6)
	Widowed	10 (20)	13 (19)
Occupation	Farmer	42 (84)	56 (81)
	Livestock keeper	0 (0)	1 (1)
	Unemployed	5(10)	12 (17)
	Other	3 (6)	0 (0)
Education level	No formal education	29 (58)	36 (52)
	Primary	20 (40)	30 (43)
	Secondary	1 (2)	3 (4)
	Tertiary	0 (0)	0 (0)

### Comparison of hematological parameters associated with podoconiosis

Samples from both cases and controls underwent hematological testing. Before comparison, data were checked for normality using the Shapiro-Wilk test. WBC, LYM, and PDW were found to be normally distributed, while all other parameters were not normally distributed and therefore required non-parametric tests. Group comparisons were conducted using independent samples t-tests for normally distributed variables (Table 2), while the Mann–Whitney U test was applied for non-normally distributed data (Table 3). Effect sizes for non-parametric comparisons were calculated using the rank-biserial correlation coefficient (r).

**Table 2:** Comparison of means between the case and control groups.

Parameter	Control Mean ± SD	Case Mean ± SD	*p*-value (t-test)	95% Confidence Interval of the Difference		Adjusted p-value (FDR)
				Lower	Upper	
WBC (10^9^/l)	4.69 ± 1.11	4.85 ± 1.00	0.416	-.55166	.22982	0.555
Lymphocytes (%)	45.84 ± 8.49	40.96 ± 10.63	0.006*	1.40625	8.35697	0.029*
PDWs (fL)	9.42 ± 1.39	8.83 ± 1.33	0.022*	.0867	1.0964	0.088

WBC = White Blood Cell, PDWs = Platelet Distribution Width.

**Table 3:** Comparison of medians between the case and control groups.

Parameter	Control’s median	Case’s median	p-value (Mann–Whitney U)	Adjusted p-value (FDR)	Effect size (r)
Lymphocytes (10^9^/l)	2.060	1.975	0.147	0.235	0.133
Monocytes (10⁹/l)	0.400	0.435	0.094	0.171	0.153
Granulocytes (10^9^/l)	2.050	2.290	0.042*	0.144	0.186
Neutrophil (10^9^/l)	1.900	2.1	0.164	0.252	0.128
Eosinophil (10^9^/l)	0.715	0.034	0.757	0.824	0.028
Basophil (10^9^/l)	0.000	0.000	0.580	0.707	0.173
Monocytes (%)	8.600	9.150	0.172	0.258	0.125
Granulocytes (%)	45.100	48.550	0.062	0.124	0.171
Neutrophil (%)	42.300	43.900	0.230	0.328	0.110
Eosinophil (%)	2.000	0.850	0.706	0.806	0.035
Basophil (%)	0.000	0.000	0.060	0.124	0.173
RBC (10¹²/l)	4.900	4.650	0.005*	0.030*	0.257
HGB (g/dl)	14.500	13.750	0.000*	<0.001*	0.364
HCT (%)	41.140	39.765	0.001*	0.012*	0.296
MCV (fl)	86.000	86.00	0.976	0.976	0.003
MCH (pg)	30.300	29.700	0.210	0.320	0.115
MCHC (g/dl)	35.200	34.700	0.002*	0.016*	0.290
RDWs (fl)	50.000	50.800	0.054	0.124	0.177
RDWc (%)	12.900	12.900	0.314	0.431	0.092
Platelets (×10^9^/l)	282.00	293.000	0.085	0.159	0.158
PCT (%)	0.190	0.190	0.471	0.628	0.066
MPV (fl)	6.800	6.600	0.042*	**0.144**	0.187

RBC = Red Blood Cell, HGB = Hemoglobin, HCT = Hematocrit, MCV = Mean Corpuscular Volume, MCH = Mean Corpuscular Hemoglobin, MCHC = Mean Corpuscular Hemoglobin Concentration, Rdws = Red Cell Distribution Width – standard devation, Rdwc = Red Cell Distribution Width- coefficient of variation, PCT = Platelecrit, MPV = Mean Platelet Volume

Raw p-values were adjusted using the Benjamini–Hochberg false discovery rate correction to account for multiple comparisons (n = 24 tests). Adjusted p-values < 0.05 were considered statistically significant.

No significant difference was observed in total white blood cell count between cases and controls (p = 0.416). However, relative lymphocyte percentage was significantly lower in podoconiosis cases compared to controls (p = 0.006). Red blood cell parameters showed significant reductions in patients with podoconiosis, including RBC count (p = 0.005, r = 0.257), hemoglobin (p < 0.001, r = 0.364), hematocrit (p = 0.001, r = 0.296), and MCHC (p = 0.002, r = 0.290), indicating small-to-moderate effect sizes. Absolute granulocyte counts were also higher in cases (p = 0.042, r = 0.186), although the effect size was small. Platelet-related parameters showed modest differences, including lower mean platelet volume (p = 0.042, r = 0.187) and platelet distribution width (p = 0.022).To account for multiple comparisons across hematological parameters, p-values were adjusted using the Benjamini–Hochberg false discovery rate correction. After adjustment, red blood cell parameters, including RBC count (adjusted p = 0.030), hemoglobin (adjusted p < 0.001), hematocrit (adjusted p = 0.012), and MCHC (adjusted p = 0.016), remained significantly lower in podoconiosis patients compared to controls. Relative lymphocyte percentage also remained significantly reduced in cases (adjusted p = 0.029). However, differences in platelet distribution width, mean platelet volume, and granulocyte counts were no longer statistically significant after correction.

### Morphological analysis of peripheral blood cells in study participants

**Fig 1:**
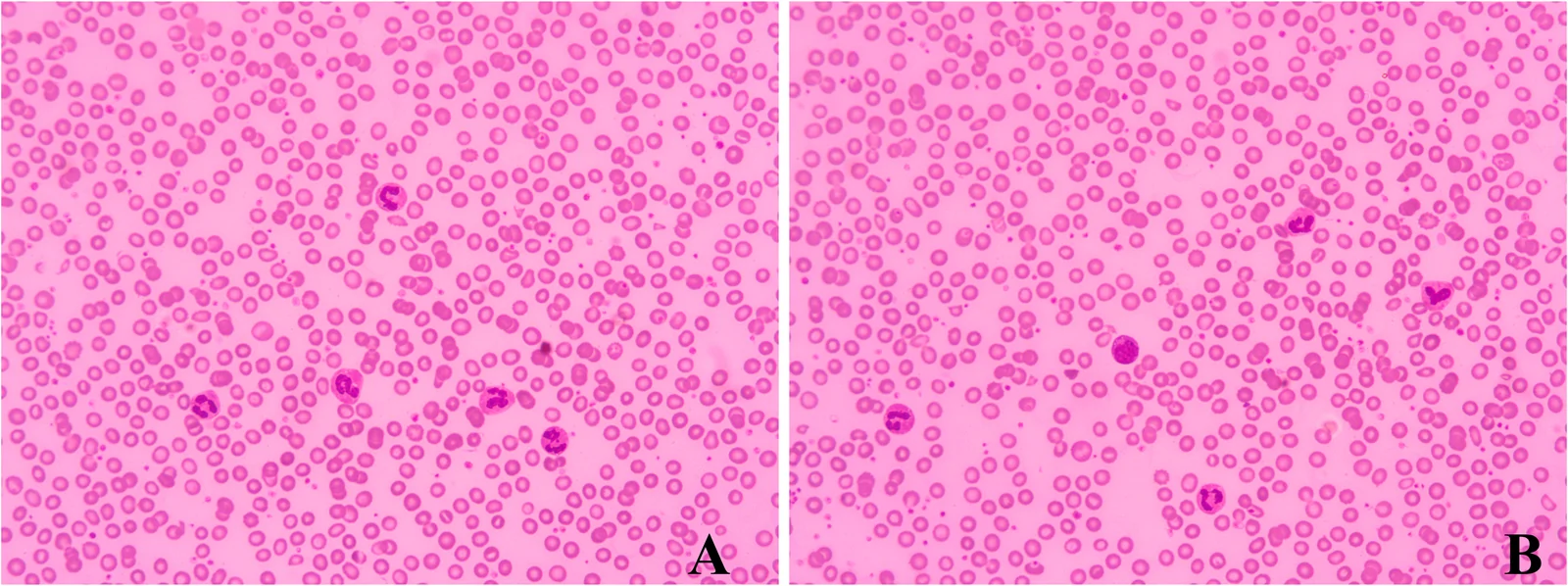
Representative peripheral blood smear of study participants. (A) presents a healthy control smear. (B) indicates a representative smear of a patient with podoconiosis.

Peripheral blood smear (PBS) examination was conducted to evaluate the morphological characteristics of blood cells in podoconiosis patients compared to healthy controls. It revealed features that were consistent with the hematological profile observed in the full blood count results. Fig 1 illustrates a typical smear presentation observed in the majority of cases. Generally, red blood cells exhibited normocytic, normochromic morphology with well-preserved structure in 45/50 (90%) of cases. Both images show predominantly normocytic and normochromic red blood cells with generally preserved size, shape, and central pallor, without evident poikilocytosis or abnormal leukocyte and platelet morphology. Mild anisocytosis was observed in 5/50 (10%) of cases. Additionally, blood smears from both groups showed normal morphology of platelets and white blood cells in 100% of participants, with no signs of toxic granulation, dysplasia, or abnormal cellular characteristics.

### The influence of knowledge and preventive practices

Data reveal that cases had lived in the area for a longer time (mean = 37.8 ± 16.9 years) compared to controls (mean = 24.6 ± 12.6 years). In cases, only 4% reported always wearing shoes, compared to 23.2% of controls. Conversely, none of the controls reported never wearing shoes, whereas 24% of the cases did. Although 68.1% of controls wore open shoes, only 14 podoconiosis cases were reported. In addition, foot hygiene practices were generally poor in both groups, with only 12% of cases and 18.8% of controls reporting regular foot washing. Referring to knowledge and beliefs, 36% of cases reported not knowing the cause of podoconiosis, and 74% did not believe barefoot exposure was a risk compared to just 11.6% and 55.1% among controls, respectively. In addition, only 60% of cases reported a lack of knowledge about preventive measures (Table 4).

**Table 4:** Podoconiosis preventive practices.

Predictor	Category	Cases (%)	Control (%)
Years living in the area	2-20	9 (18)	27 (39.1)
	21-40	23 (46)	37 (53.6)
	41-60	13 (26)	3 (4.34)
	61-80	5 (10)	2 (2.8)
Wearing shoes regularly	Always	2 (4)	16 (23.2)
	Sometimes	20 (40)	49 (71.0)
	Rarely	16 (32)	4 (5.8)
	Never	12 (24)	0 (0.0)
Type of shoe wearing	Always barefoot	14 (28)	0 (0.0)
	Open shoes	7 (14)	47 (68.1)
	Plastic shoes	29 (58)	14 (20.3)
	Closed shoes	0 (0)	8 (11.6)
Regular foot hygiene	No	44 (88)	56 (81.2)
	Yes	6 (12)	13 (18.8)
Knowledge on podoconiosis causes	Inherited	19 (38)	29 (42.0)
	Poor hygiene	2 (4)	3 (4.3)
	Types of soil	8 (16)	22(31.9)
	Virus or bacteria	3 (6)	7 (10.1)
	I don’t know	18 (36)	8 (11.6)
Believing barefoot causes podoconiosis	No	37 (74)	38 (55.1)
	Yes	1 (2)	23 (33.3)
	Not sure	12 (24)	8 (11.6)
Knowledge on prevention	No	30 (60)	62 (89.9)
	Yes	20 (40)	7 (10.1)

### Clinical pathological features of podoconiosis patients

**Fig 2:**
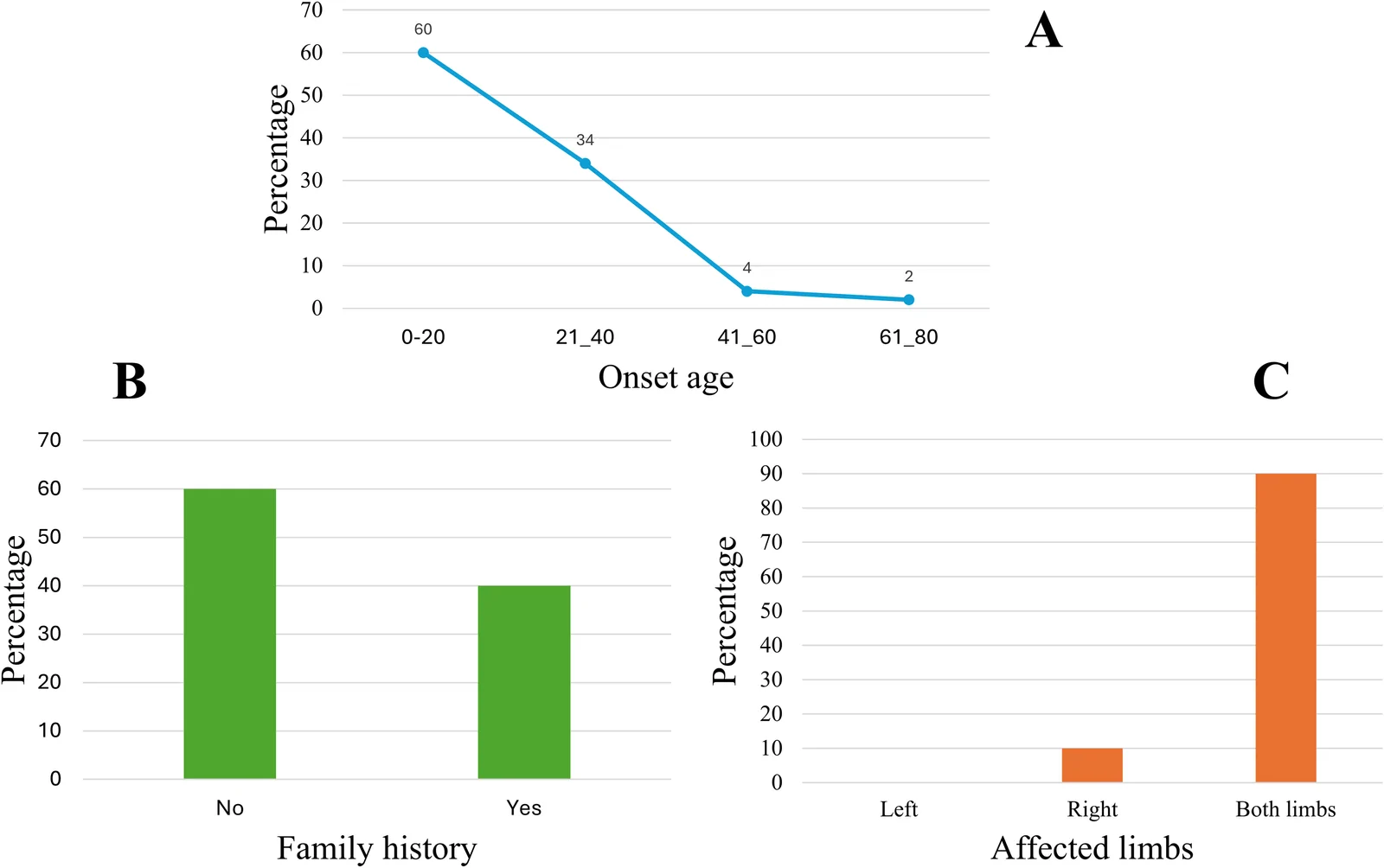
Clinical pathological features. (A) Age of onset distribution. (B) Family history of the condition. (C) Distribution of affected limbs.

Factors such as age of onset, laterality of limb involvement, and family history contribute to understanding the age groups most at risk, potential genetic predispositions, and patterns of disease manifestation. Fig 2 illustrates the study findings related to these key factors. The distribution of onset age among podoconiosis patients ranged from 4 to 65 years, with a mean onset age of 21.06 ± 13.15 years, with the majority (60%) developing symptoms between ages 0–20 years. Regarding limb involvement, 90% of patients presented with bilateral leg swelling. Potential familial clustering was suggested by the fact that 40% of podoconiosis patients in the current study reported having a family member who had previously had the illness.

## Discussion

This study aimed to identify hematological profile and factors that contribute to a better understanding of podoconiosis risk and disease impact. The findings provide important insights into hematological profiles, sociodemographic factors, and behavioral risks associated with the disease.

The gender distribution shows that the majority of participants were female (84%) in both groups, which aligns with previous research in Rwanda [9] and in Ethiopia [18], both reported a higher prevalence of podoconiosis among women. This trend is often linked to greater exposure to irritant soil during farming and limited use of protective footwear, which may disproportionately affect women. However, [17] found more male cases (64.2%) in their small hospital-based sample, likely reflecting sampling methods rather than true population trends [17]. Regarding age distribution, most of the podoconiosis patients were older compared to the control; similar observations were reported, indicating that podoconiosis tends to affect older individuals due to cumulative exposure over time [17,18].

Most participants were married, with a notable proportion being divorced or widowed, which may reflect the social stigma and isolation associated with the disease. This aligns with another study, which highlighted reduced social support among podoconiosis patients [19]. Occupationally, the vast majority (84%) were subsistence farmers, reinforcing the well-established association between barefoot farming and podoconiosis. Additionally, low educational attainment was common, with 58% of patients and 52% of controls having no formal education. This finding is consistent with other studies reporting that most podoconiosis patients lacked formal schooling [9,17,19]. Thus, a lack of education may hinder awareness of preventive measures and increase susceptibility to the disease.

Findings about the hematological parameters reveal that the relative lymphocyte count was significantly reduced in the podoconiosis group. In contrast, the absolute lymphocyte count did not show a significant difference between groups, which may represent a relative shift in distribution rather than true lymphopenia. Additionally, granulocyte absolute counts were significantly higher in cases but this difference was no longer statistically significant after adjustment for multiple comparisons (adjusted p = 0.144), suggesting a trend rather than a definitive change. Since investigations showed that podoconiosis is characterised by enhanced innate immune activity, these findings point to a shift in leukocyte distribution toward greater innate immune activity in podoconiosis [9]. Despite non-significant changes in the individual granulocyte subtypes (neutrophils, eosinophils, and basophils), the observed trend in the total granulocyte count presumably represents modest, cumulative elevations across the granulocyte lineages, suggesting a subclinical inflammatory state rather than a shift restricted to a single lineage. This pattern is consistent with the observation that even mild increases in leukocyte populations can signal early systemic immune activation and low-grade inflammation [20]. However, the interpretation of lymphocyte and granulocyte findings should also consider the age imbalance. Aging is associated with immunosenescence and changes in leukocyte distribution, including increased neutrophil counts and neutrophil-to-lymphocyte ratio, as well as age-related changes in lymphocyte counts. Therefore, the lower lymphocyte percentage and the trend toward higher granulocyte counts observed among cases may be partly influenced by age-related immune changes rather than podoconiosis alone. In contrast, a different study reported a decrease in total white blood cell count, neutrophils, and eosinophils, accompanied by increased lymphocytes and monocytes [17].

Although red cell indices (RBC, HGB, HCT, and MCHC) were statistically significant with small-to-moderate effect sizes (r = 0.257–0.364), the values for both groups remained within normal physiological ranges. This indicates that red cell indices are not overtly abnormal in podoconiosis patients. However, there may be a subtle subclinical effect of chronic inflammation (due to chronic exposure), and these findings are consistent with the early stages of ACD, as supported by the literature. It is known that ACD may initially show normal red cell indices before developing into overt anemia [21]. In line with a study conducted in Ethiopia, podoconiosis patients had significantly decreased hemoglobin and red cell indices, while values were still close to normal ranges, suggesting borderline anemia [17]. Notably, there was no significant difference in mean corpuscular hemoglobin (MCH) or mean corpuscular volume (MCV) between the groups (p > 0.05), indicating that the anemia is caused by cytokine-mediated marrow suppression, which is a feature of ACD, rather than a nutritional deficiency (such as iron or B12).

Despite the fact that mean platelet volume (MPV) and platelet distribution width (PDW) were lower in cases, these differences were no longer statistically significant after FDR adjustment (MPV adjusted p = 0.144, PDW adjusted p = 0.088), suggesting a trend rather than a definitive change. These observations may reflect subtle platelet activation or increased platelet consumption, potentially reflecting chronic low-grade inflammation. It was described how platelets actively participate in inflammation through the release of interleukin-1β and chemokines, promoting vascular permeability and endothelial dysfunction [22]. The study conducted in Cameroon by Ndzeshang et al. observed that although mean platelet indices appeared within normal ranges, a substantial proportion of podoconiosis patients exhibited abnormal values, 47.7% for MPV, 66.2% for PDW, and 58.1% for plateletcrit (PCT). These results indicate considerable platelet heterogeneity within the podoconiosis population, with evidence of platelet activation even in the absence of elevated total counts [10].

Microscopic results imply that, despite its chronic inflammatory nature, podoconiosis in its uncomplicated form does not cause overt morphological changes in circulating cells. This finding aligns with the study, which reported that cellular morphology remained largely preserved in podoconiosis patients, despite quantitative variations in hematological indices [10]. These observations strongly support the hypothesis that podoconiosis predominantly affects lymphatic and immunologic pathways rather than directly disrupting hematopoietic cellular structure.

Podoconiosis is considered a multifactorial disease, including environment and behavioral aspects. In this study, it was observed that cases had lived in the area significantly longer than controls suggests a possible cumulative environmental exposure contributing to disease development. This finding is similar to the previous studies, which reported that endemicity in podoconiosis-affected regions is strongly linked to long-term residency in high-risk zones [9]. Findings showed that among podoconiosis cases, only 4% always wear shoes, compared to 23.2% of controls. This highlights the recognised benefit of consistently wearing shoes as a primary preventive measure against podoconiosis. The cornerstone of prevention is wearing footwear that protects individuals from direct contact with irritant soil [4]. Similarly, Tora et al. found that the lack of protective footwear among rural children significantly increased the vulnerability to podoconiosis [23].

None of the controls went barefoot, although a significant percentage of patients (28%) did. The fact that plastic shoes frequently offer insufficient protection, cases were more likely to wear them (58%) than controls (20.3%). Additionally, in contrast to 11.6% of controls, none of the patients wore closed shoes. This indicates that open or plastic footwear (non-protective shoes) provides inadequate protection and that social and financial obstacles frequently make it impossible to wear appropriate shoes regularly. Findings also reported significant differences between cases and controls in shoe-wearing history and the type of shoes worn [17]. Foot hygiene was generally poor across both groups, with low rates of regular foot washing reported.

Even if shoe-wearing and foot hygiene are the key factors to the condition, a factor alone may not be sufficient to prevent podoconiosis unless combined with other preventive measures completely. It was reported that people who did not routinely wash their feet with soap and water in sub-Saharan Africa had a 2.8-fold higher risk of developing podoconiosis (95% CI: 1.16–4.44, p = 0.001). In comparison, barefoot walkers had a 5.7-fold higher risk [24]. These results highlight how important it is to maintain good foot cleanliness and wear shoes consistently in order to prevent podoconiosis. These findings should also be interpreted within the broader context of podoconiosis prevention and care in Musanze District. Previous research reported that the implementation of podoconiosis-related interventions in the district is influenced by geographical, socioeconomic, sociocultural, and health-system factors, including hilly terrain, long travelling distances, financial constraints, stigma, gender-related discrimination, and dependence on externally supported programmes [25]. These contextual barriers may limit access to appropriate footwear, regular attendance at treatment centres, and adherence to recommended preventive practices. Nevertheless, locally implemented health-promotion initiatives have contributed to improved community awareness and earlier care-seeking, highlighting the importance of combining individual preventive education with interventions that address structural and social barriers.

The results of this study reveal a substantial knowledge gap that likely contributes to risky behaviors. In a similar vein, Yakob et al. discovered that significant misunderstandings about the causality of the disease in the community may delay prevention efforts [26]. Clearly, 74% of cases did not believe barefoot causes the disease, despite its well-established epidemiological association. Accordingly, there is a lack of knowledge among communities and health professionals [27]. Remarkably, compared to 89.9% of controls, 60% of cases reported a lack of knowledge about prevention. Yet, as pointed out, knowledge alone doesn’t translate into safer behavior without addressing practices like shoe-wearing and other preventive strategies [28].

The distribution of onset age among podoconiosis patients reveals that the majority (60%) developed symptoms between the ages of 0–20 years, with an additional 34% affected between the ages of 21–40 years, indicating that onset commonly occurs within the first decades of life. This early onset highlights the importance of initiating preventive measures during childhood and adolescence, such as educating children about proper foot hygiene and consistent shoe-wearing. These findings are partially in line with those of Bikorimana et al., who reported a mean onset age of 34.4 years, with a significant proportion of patients developing the disease before the age of 20 [29]. Similarly, it's noted that the average age at which leg swelling is first noticed is around 25 years, with the disease remaining common into the sixth decade of life [4].

The majority of patients presented with bilateral leg swelling, consistent with the clinical hallmark of podoconiosis, which typically presents as bilateral but asymmetric lower limb swelling and is often used to distinguish it from other causes of lymphoedema such as filariasis, which may present unilaterally [30,31]. The absence of left-only cases further supports the tendency toward bilateral progression. Only 40% of podoconiosis patients in the current study reported having a family member who had previouslyhad the illness [25]. In comparison, Davey et al. found a sibling recurrence risk ratio of 5.07 and heritability estimated at 63% [30]. Furthermore, it has also been reported that 51% of patients in Rwanda had a family history [29].

These reports highlight the significance of gene–environment interactions in the contribution to podoconiosis. However, the fact that 60% of patients in the present study had no known family history may highlight that environmental exposure and individual characteristics remain a critical driver of disease onset, emphasising the multifactorial nature of podoconiosis and the need for prevention strategies that address both genetic susceptibility and modifiable environmental risks.

The study has some limitations that should be considered when interpreting the findings. Participants were recruited using convenience sampling, which may affect representativeness and generalizability. Additionally, although all participants were adults aged 18 years and above, cases were generally older than controls, and no formal age matching or statistical adjustment was performed, which could influence hematological comparisons. The severity stages of podoconiosis were also not assessed or taken into consideration during the analysis, limiting the ability to determine whether hematological parameters varied according to disease severity. Lastly, factors such as biochemical and immunological profiles were not assessed, indicating opportunities for future research.

## Conclusion

This study found that patients with podoconiosis had poorer footwear habits, limited knowledge of disease causes and prevention, and significant changes in hematological parameters, including lower red blood cell indices, lymphocyte and platelet indices, and higher granulocyte counts. These differences most likely reflect chronic immunological activation and point to the presence of subclinical inflammation, which may have an impact on the course of the disease and general health. The results underscore the necessity of integrated prevention measures centered on community education, continuous protective behavior promotion, and early hematological monitoring. Furthermore, future research should encompass multiple regions and assess unexplored biological markers to enable the development of more effective and specific management strategies.

## Abbreviations

CI: Confidence Interval
Hb: Hemoglobin
HASA: Heart and Sole Action
HCT: Hematocrit
INES: Institut d’Enseignement Supérieur de Ruhengeri
LMICs: Low- and Middle-Income Countries
MCH: Mean Corpuscular Hemoglobin
MCHC: Mean Corpuscular Hemoglobin Concentration
MCV: Mean Corpuscular Volume
MPV: Mean Platelet Volume
PCT: Plateletcrit
PLT: Platelets
RBC: Red Blood Cells
RDW: Red Cell Distribution Width
SPSS: Statistical Package for the Social Sciences
WBC: White Blood Cells
WHO: World Health Organization
